# The Association Between Central Venous Pressure and Acute Kidney Injury Development in Patients with Septic Shock †

**DOI:** 10.3390/jcm14093027

**Published:** 2025-04-27

**Authors:** Nasrin Nikravangolsefid, Jacob Ninan, Supawadee Suppadungsuk, Waryaam Singh, Kianoush B. Kashani

**Affiliations:** 1 Division of Nephrology and Hypertension, Department of Medicine, Mayo Clinic, Rochester, MN 55905, USA; nasrinnik67@gmail.com (N.N.); supawadee.sup@mahidol.ac.th (S.S.); singh.waryaam@mayo.edu (W.S.); 2 Division of Pulmonary and Critical Care Medicine, Department of Medicine, Mayo Clinic, Rochester, MN 55905, USA; ninan.jacob@outlook.com; 3 Department of Nephrology and Critical Care, MultiCare Capital Medical Center, Olympia, WA 98502, USA; 4 Chakri Naruebodindra Medical Institute, Faculty of Medicine Ramathibodi Hospital, Mahidol University, Samut Prakan 10540, Thailand

**Keywords:** acute kidney injury, septic shock, sepsis, central venous pressure

## Abstract

**Background:** Sepsis-associated acute kidney injury (AKI) is linked to increased mortality and prolonged hospital stays. The exact relationship between central venous pressure (CVP) and AKI remains unclear. We explored the correlation between CVP and AKI in septic shock patients. **Methods:** This retrospective study included adult patients with septic shock admitted to Mayo Clinic Rochester between 2006 and 2018. CVP levels were measured at 6, 12, 24, and 48 h after the diagnosis of sepsis, and patients were stratified into two groups based on CVP levels (CVP < 8 or ≥8 mmHg). **Results:** Of 5600 patients with septic shock, 3128 patients without AKI on admission are included. One-thousand-and-ninety-eight patients (35.1%) developed AKI within a median of 4.4 days. The median CVP levels and frequency of elevated CVP at 6, 12, 24, and 48 h are significantly higher in the AKI group. Elevated CVP (≥8 mmHg) at 6, 12, 24, and 48 h is associated with AKI incidence, even after adjusting for mean arterial pressure (MAP) levels. This association, after multivariable adjustments, only remains significant at 12 h with an odds ratio (OR) of 1.60 (95% CI, 1.26–2.05), *p* < 0.001 and 48 h with an OR of 1.60 (95% CI, 1.29–1.99), *p* < 0.001. **Conclusions:** Our findings indicate that CVP ≥ 8 mmHg is strongly associated with an increased risk of AKI, even after adjusting for MAP at the 12 and 48 h time points. These findings underscore a critical 12 or 48h window for interventions to lower CVP.

## 1. Introduction

Septic shock is associated with an average 30-day mortality rate of 35% [1,2]. Among the organ dysfunctions associated with septic shock, acute kidney injury (AKI) is common, affecting up to 60% of septic shock patients within 24 h following the onset of hypotension [3]. Patients with septic shock who develop AKI experience significantly higher mortality rates and more extended hospital days compared to those without AKI [3,4,5].

Central venous pressure (CVP) is an indicator of right atrial pressure and is often used as a predictor of right ventricular end-diastolic volume, also known as its preload. [6,7]. The mean CVP at 12–24 h in the EGDT [8], ARISE [9], ProCESS [10], and ProMISE [11] trials, which greatly influenced the Surviving Sepsis Guidelines, was in the range of 11–11.9 mmHg. Although used as a primary metric for fluid therapy, the latest Surviving Sepsis Guidelines no longer recommends CVP as a tool for fluid resuscitation, instead emphasizing the maintenance of the mean arterial pressure (MAP) of ≥65 mmHg to ensure adequate organ perfusion [12,13]. A pressure gradient between the arterial and the venous pressure maintains tissue perfusion. MAP determines the forward flow of blood into organs and is correlated with cardiac output. CVP surrogates the venous system capacity, stressed volume, and pressures on the right side of the heart [14,15]. Although resuscitation strategies often focus on forward determinants of kidney blood flow, such as MAP, the backward elements, like CVP, are less well-studied.

High CVP levels are typically associated with fluid overload or decreased cardiac capacity, which may lead to kidney congestion [16,17]. Yet, the precise relationship between CVP values and AKI incidence is not well-defined in the current literature. This study aimed to investigate the relationship between CVP thresholds and the development of AKI in patients with septic shock.

## 2. Materials and Methods

### 2.1. Study Setting and Population

In this historical cohort study, we included adult patients (≥18 years old) admitted with septic shock to Mayo Clinic intensive care units between August 2006 and March 2018. Septic shock was defined based on the third international consensus (Sepsis-3) definition [18]. This study was approved by the Institutional Review Board of Mayo Clinic (IRB ID: 20-009394), and obtaining informed consent for patients with the Minnesota research authorization was waived due to the minimal-risk nature of this study.

We excluded patients with end-stage kidney disease (ESKD) on kidney replacement therapy, those with a baseline creatinine level above 4 mg/dL, individuals who died or were discharged within the first 48 h of admission, and patients who presented with AKI on admission. We also excluded individuals who did not have recorded central venous pressure (CVP) measurements.

### 2.2. Data Collection and Variables

The Anesthesia Clinical Research Unit (ACRU) extracted the data from the Mayo Clinic ICU datamart. We collected demographics (age, sex, and body mass index [BMI]), comorbidities), the severity of illness scores (Acute Physiology, Age, and Chronic Health Evaluation [APACHE] III within 1–24 h of admission), hemodynamic parameters (mean arterial pressure [MAP], and CVP at four time points, i.e., 6, 12, 24, and 48 h post-sepsis diagnosis), and net fluid balance within 24 h, laboratory parameters (baseline serum creatinine [Cr], Cr level close to ICU admission, baseline estimated glomerular filtration rate [eGFR], maximum Cr before AKI, white blood cell count [WBC], and lactate levels). Although some data were automatically recorded in the patient’s electronic records from the patient monitors, perfusion pumps, and mechanical ventilators, nurses also manually recorded hemodynamic parameters (like heart rate, blood pressure, MAP, and CVP), and urine output. Only manually validated data were extracted for analysis. We also abstracted the data regarding sepsis management, including the use of mechanical ventilation, average positive end-expiratory pressure (PEEP), use of dialysis in the ICU and hospital, and vasopressors (including dobutamine, dopamine, epinephrine, norepinephrine, phenylephrine, and vasopressin within 24 h of ICU admission).

### 2.3. Definitions and Outcomes

Our outcomes included acute kidney injury (AKI), as defined by an increase in serum creatinine to ≥1.5 times the baseline level within seven days (modified KDIGO [19] AKI definition by excluding a serum creatinine increase of greater than 0.3 mg/dL and decrease in urine output) as as well as in-hospital, 28-, and 90-day mortality rates. We stratified patients into two groups based on their CVP measures (<8 mm Hg or ≥8 mm Hg) at the previously specified time points, i.e., 6, 12, 24, and 48 h after sepsis diagnosis. The early goal-directed trial (EGDT) aimed to maintain CVP in the range of 8–12 mm Hg, and since all the earlier-mentioned trials attempted to replicate the EGDT, we aimed for the lower level of this CVP range. Mean perfusion pressure (MPP) [20,21,22,23] was calculated as the difference between MAP and CVP.

### 2.4. Statistical Analysis

Statistical analysis was conducted using SPSS (version 22, IBM Corp., Armonk, NY, USA), with the chi-squared test applied to categorical variables, reported as numbers (percentage). For continuous variables, the Mann–Whitney U test and the Kruskal–Wallis test were used, with the results presented as the median and interquartile range (IQR). Multivariate logistic regression analysis was employed to determine the associations between CVP levels and primary outcomes at the prespecified time points. Multiple Cox regression analysis was utilized to determine the associations between CVP levels and 28-day and 90-day mortality in patients with septic shock. Kaplan–Meier survival curves were created to compare the survival rates between AKI and CVP groups (<8 mmHg or ≥8 mmHg). All logistic and Cox regression results are reported as odds ratios (ORs) or hazard ratios (HRs), along with their 95% confidence intervals (CIs). A *p*-value of <0.05 was considered statistically significant.

## 3. Result

### 3.1. Patient Characteristics

**Figure 1:**
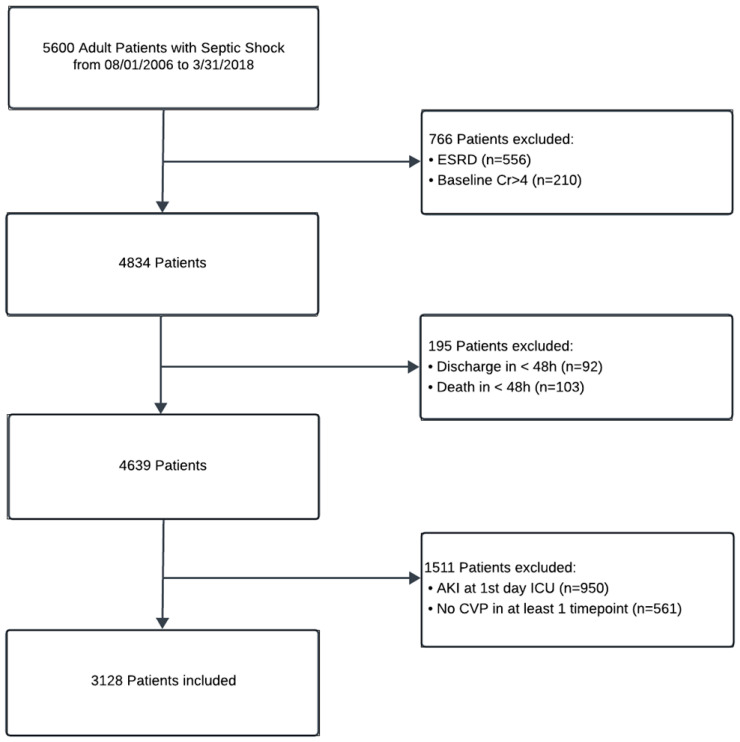
Flowchart of patient inclusion in this study.

**Figure 2:**
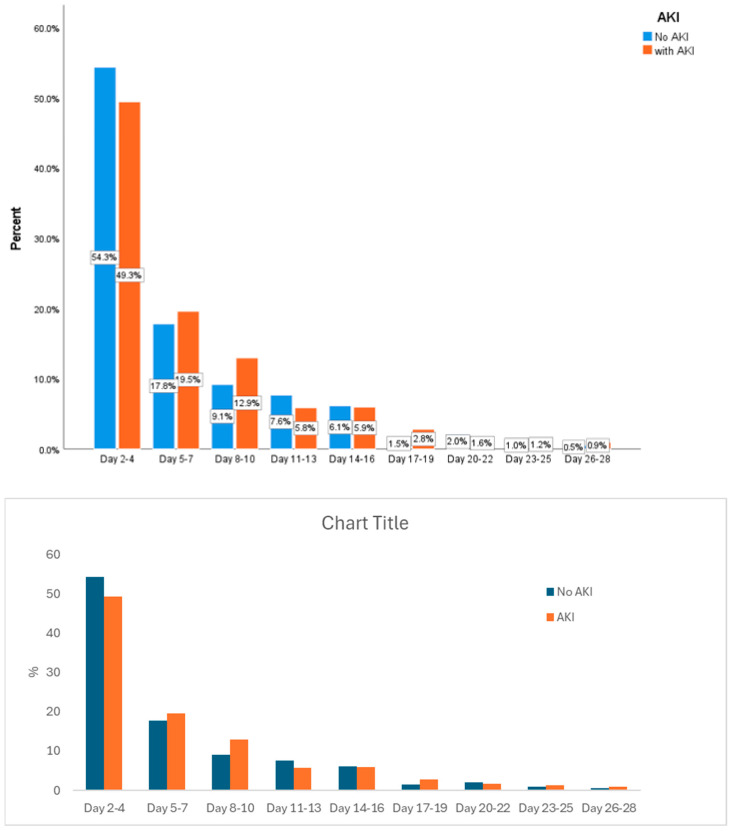
Incidence of new AKI development after ICU admission.

Out of 5600 screened patients with septic shock, 3128 eligible patients without AKI on the ICU admission day were included in this study (Figure 1). The median age was 65.5 years (IQR: 55–75), and 1681 (53.7%) were male. Of these, 1098 patients (35.1%) developed AKI within a median of 4.4 days (2–9) following admission, including 375 (34.15%) with AKI stage 1, 399 (36.34%) with AKI stage 2, and 324 (29.51%) with AKI stage 3. The majority of sepsis-associated AKI (49.3%) developed between the second and fourth days of ICU admission (Figure 2). Table 1 shows the characteristics of included patients and those with and without AKI.

In the AKI group, male sex, history of hypertension, diastolic heart failure, and the use of vasopressin, norepinephrine, and epinephrine were significantly higher. The median age and frequency of other comorbidities, such as diabetes mellitus, myocardial infarction, congestive heart failure, pulmonary hypertension, and mild-to-moderate chronic kidney disease, did not statistically differ between the AKI and non-AKI groups. The incidence of cerebrovascular accidents (CVAs) was lower in the AKI group (*p* < 0.001). The APACHE III score within the first 24 h of ICU admission was significantly higher in the AKI group (*p* < 0.001).

Baseline creatinine levels were higher in the non-AKI group (0.93 vs. 0.90, *p* < 0.001), while the creatinine levels close to ICU admission and maximum creatinine levels before the onset of AKI were significantly higher in the AKI group (1.40 vs. 0.80 mg/dL, *p* < 0.001 and 1.60 vs. 1.30 mg/dL, *p* < 0.001). The median age (65.0 vs. 65.6 years, *p*: 0.3) and WBC count close to ICU admission (10.5 vs. 10.8 K/µL, *p*: 0.2), and maximum WBC before AKI onset (12.6 vs. 13.4 K/µL, *p*: 0.2) were not statistically different between the groups. The lactate levels were significantly higher in the AKI group (2.00 vs. 1.70 mmol/L, *p* < 0.001). Also, net fluid balance within the first day of ICU was significantly higher in the AKI group (4834 vs. 4382 mL, *p*: 0.002).

The use of mechanical ventilation and the median values of PEEP were higher in the AKI group. Among the AKI patients, 28.7% and 31.6% underwent renal replacement therapy during their ICU and hospital admission, respectively. Furthermore, the 28- and 90-day mortality rates and the duration of hospital stay were higher in the AKI group (*p* < 0.001).

### 3.2. CVP at Different Time Points and AKI

The median CVP levels at 6, 12, 24, and 48 h after sepsis diagnosis were significantly higher in AKI groups (10 vs. 8, 10 vs. 7, 10 vs. 8, 10 vs. 8 mmHg, respectively, *p* < 0.001). Similarly, the frequency of CVP level ≥ 8 mmHg at all measured time points was also significantly higher in the AKI groups (40.5% vs. 36.7%, 46.5% vs. 39.4%, 56.3% vs. 45.7%, 62.3% vs. 48.1%, respectively, *p* < 0.001). The comparison of CVP, MPP, MAP levels, and frequency of elevated CVP between patients with and without AKI in the four time points after the diagnosis of sepsis is presented in Table 2. The percentage of patients achieving a MAP goal of ≥65 mmHg was not significantly different between the AKI and non-AKI groups at 6 h (68.9% vs. 71.5%, *p*: 0.111). However, it was notably lower at 12, 24, and 48 h following sepsis diagnosis in the AKI groups (70.8% vs. 77.3%, 74.4% vs. 80.1%, 80.9% vs. 85.6%, respectively, *p* < 0.001).

Logistic regression analysis showed that elevated CVP (≥8 mmHg) was associated with a higher risk of AKI with an odds ratio (OR) of 1.56 (95% CI: 1.30–1.89, *p* < 0.001). This relationship persisted after adjusting for MAP at 6, 12, 24, and 48 h (1.61, 95% CI: 1.33–1.94; 1.81, 95% CI: 1.52–2.16; 1.89, 95% CI: 1.60–2.24; and 1.78, 95% CI: 1.52–2.09, respectively, *p* < 0.001). However, when further adjustments were made to include MAP ≥ 65 mmHg, age, sex, BMI, APACHE score, comorbidities, use of vasopressors, baseline creatinine, eGFR, lactate levels, average PEEP, and mechanical ventilation, the OR was no longer significant at 6 (OR: 1.20, 95% CI: 0.92–1.57, *p*: 0.173) and 24 h (1.25, 95% CI: 0.99–1.57, *p*: 0.056). Still, it remained significant at 12 and 48 h with ORs of 1.60 (95% CI, 1.26–2.05; *p* < 0001) and 1.60 (95% CI, 1.29–1.99; *p* < 0001), respectively (Table 3).

After adjusting for the multivariable, each one mmHg increase in CVP (≥8 mmHg) was related to 1.47 (95% CI: 1.08–2.01, *p*: 0.015) odds of AKI at 6 h and 1.44 (95% CI: 1.11–1.86, *p*: 0.006) odds of AKI at 48 h. These odds were not significant at 12 h and 24 h (1.20, 95% CI: 0.88–1.64, *p*: 0.248 and 1.32, 95% CI: 0.99–1.76, respectively, *p*: 0.055). In the sub-cohort of patients with pulmonary hypertension, the association of CVP in the development of acute kidney injury did not differ from the whole cohort. Adjustments for fluid balance at each point did not impact the results; therefore, it was removed from the variables that assessed the risk of AKI due to CVP.

### 3.3. Survival Analysis

**Figure 3:**
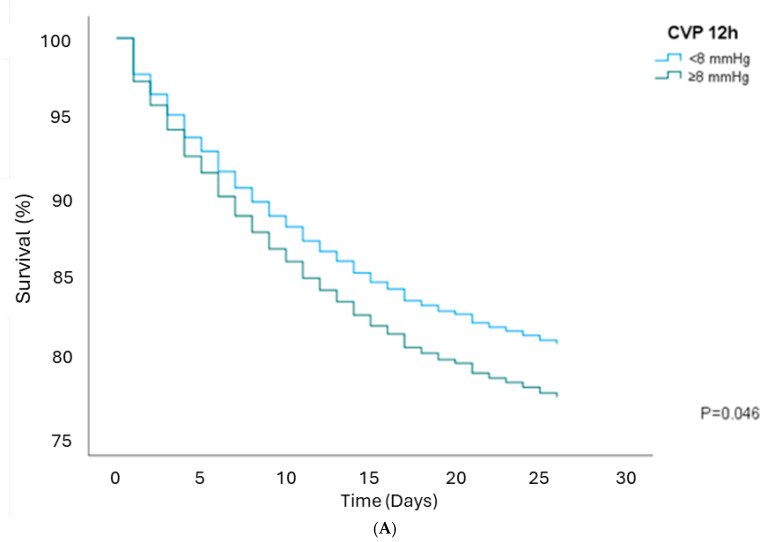

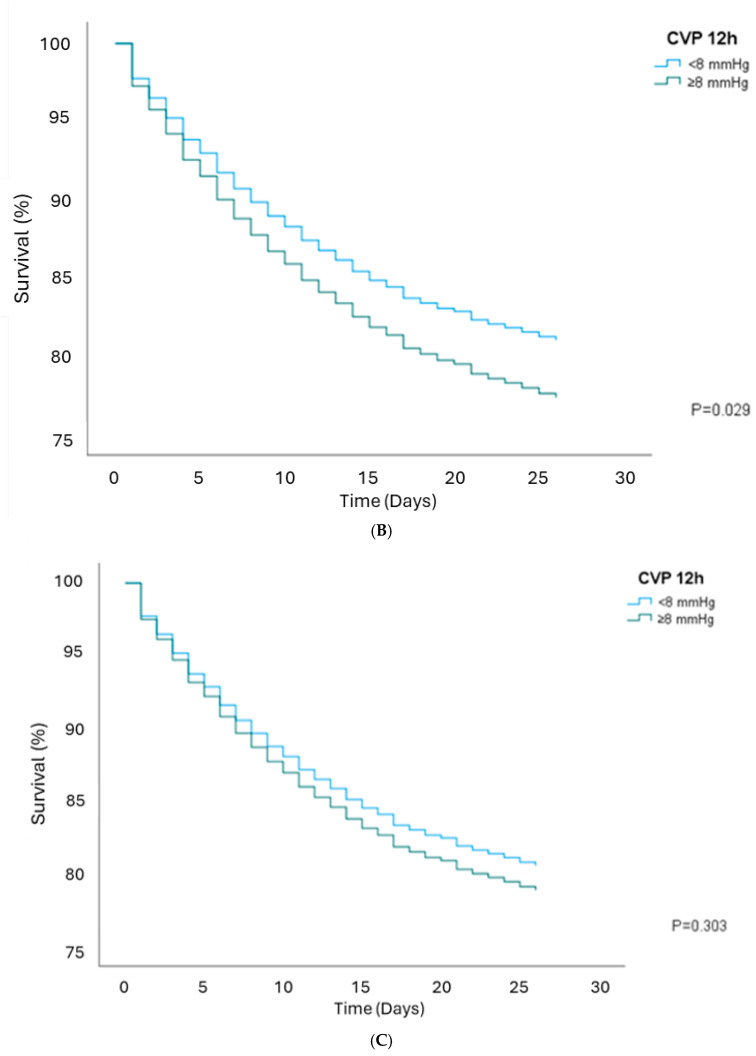

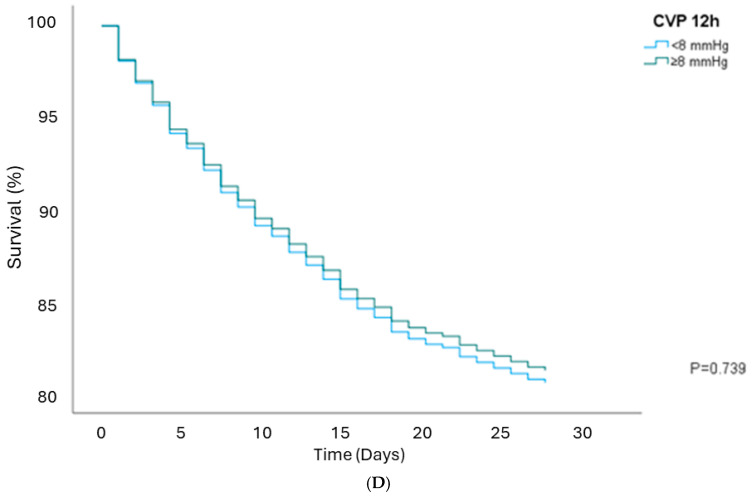
Kaplan–Meier survival curve 28-day mortality rates between CVP groups (**A**) at 12 h; (**B**) at 12 h, after adjusting for MAP ≥65 mm Hg; (**C**) at 12 h, after adjusting for AKI; and (**D**) at 12 h, after adjusting for MAP, AKI, age, sex, BMI, APACHE, comorbidities, vasopressors, baseline Cr, eGFR and lactate, net fluid balance day 1, average PEEP, and mechanical ventilation.

CVP of ≥8 mmHg was not associated with in-hospital mortality in unadjusted models or when adjusted for MAP, AKI, age, sex, comorbidities, use of vasopressors, baseline creatinine, eGFR, lactate levels, average PEEP, and mechanical ventilation, except at the 12 h time point (Table 4). At 12 h, elevated CVP was linked to higher 28-day mortality, with an HR of 1.19 (95% CI, 1.00–1.43, *p*: 0.046, Figure 3A). After adjusting for MAP, the HR was 1.22 (95% CI, 1.02–1.46, *p*: 0.029, Figure 3B). However, this association was not significant after adjusting for AKI, with an HR of 1.09 (95% CI, 0.92–1.31, *p*: 0.303, shown in Figure 3C). Additionally, after adjusting for MAP, AKI, age, sex, BMI, APACHE, comorbidities, vasopressors, baseline Cr, eGFR, lactate on the first admission day, average PEEP, and mechanical ventilation, the HR was 0.96 (95% CI, 0.76–1.21, *p*: 0.739, shown in Figure 3D). Similarly, elevated CVP at 6 h, when adjusted for MAP, was associated with higher 90-day mortality, with an HR of 1.20 (95% CI, 1.02–1.41, *p*: 0.031), but this association was insignificant after adjusting for AKI with an HR of 1.10 (95% CI, 0.93–1.30, *p*: 0.251).

The mortality rates on 28 and 90 days were significantly higher in the AKI group (31.4% and 41.4% vs. 16.7% and 25.8%, respectively). Cox regression analysis indicated that AKI alone was associated with a higher 28-day and 90-day mortality, with a hazard ratio (HR) of 2.06 (95% CI, 1.77–2.40), *p* < 0.001 and 1.83 (95% CI, 1.61–2.07), *p* < 0.001, respectively. Kaplan–Meier curves comparing 28-day and 90-day mortality between AKI and non-AKI patients are displayed in Supplementary Figures S1 and S2, respectively.

## 4. Discussion

Although the recent guidelines no longer advocate for CVP monitoring as a tool for fluid resuscitation in sepsis [12,13], the role of CVP in the development of AKI remains a subject of debate. This study highlights the relevant findings about the dynamics of CVP and MAP in patients with septic shock and their associations with the development of AKI. Notably, median CVP levels and the frequency of elevated CVP (>8 mmHg) were higher in patients with AKI at all measured time points. These elevated pressures strongly correlated with increased incidences of AKI, suggesting that increased right atrial preload, increased right ventricular afterload, or right ventricular dysfunction following sepsis might play a role in AKI development. This highlights the crucial role of hemodynamic management in septic shock patients.

Interestingly, while the initial association of elevated CVP with AKI persisted after adjusting for MAP levels, this relationship was insignificant when controlling for a comprehensive set of clinical variables, except at the 12 and 48 h time points. This finding indicates that, while CVP may be an indicator of risk for AKI, its impact can be confounded by other clinical factors during specific timelines in patient care. The significant association between CVP and AKI at 12 and 48 h suggests that elevated CVP may still be a relevant predictor of AKI development, independent of other clinical factors. Xiao et al. [24] showed that elevated CVP within 24 h was linked to the risk of AKI and highlighted the importance of managing CVP levels within the first day of septic shock, aiming for an optimal CVP in the range of 4.4–8.8 mmHg. Therefore, high CVP in this critical window can be evaluated as a potential therapeutic target to mitigate the risk of AKI in this patient population.

Several studies support our findings, including one prospective cohort by Yegenaga et al. [25] on 257 adult critically ill patients with sepsis. They found that CVP at 24 h was associated with a higher AKI incidence, with an OR of 1.50 (95% CI, 1.26–1.80) after adjusting for age, hepatic failure, vascular surgical intervention, MAP, diastolic blood pressure (DBP), urinary output, creatinine, blood urea nitrogen, GFR, pH, bicarbonate, platelet count, albumin, prothrombin time, and the use of diuretics and vasopressors. However, it is essential to note that they used a different definition for AKI adjudication, i.e., serum creatinine > 2 mg/dL or oliguria (urine output less than 400 mL/24 h), and included patients with sepsis based on the presence of systemic inflammatory response syndrome (SIRS).

Another study by Legrand et al. [26] on 137 patients with sepsis in a surgical ICU showed that CVP values were associated with the risk of new or persistent AKI, even after adjusting for fluid balance and PEEP levels, with an odds ratio of 1.22 (95% CI, 1.08 to 1.39). This suggests the role of venous congestion in the pathophysiology of AKI in post-surgical septic patients. However, this study employed the Acute Kidney Injury Network (AKIN) criteria to define AKI and was limited to surgical ICU settings. Xiao et al. [24] conducted a study using the AmsterdamUMCdb database, which included 9668 patients with septic shock, and found that high CVP was associated with AKI. This association persisted even after adjusting for age, sex, weight, height, mechanical ventilation, vasopressor use, kidney replacement therapy (KRT), SOFA score, vital signs, and other clinical indicators.

Sun et al. [16] conducted a study using the Medical Information Mart for Intensive Care (MIMIC)-III database, which included critically ill adult patients with and without sepsis. They found an association between mean CVP and the incidence of AKI, with an odds ratio of 2.80 (95% CI: 2.32–3.37) after adjusting for demographics, treatment interventions, and comorbidities. However, the study did not specify whether patients with AKI at admission or those with a history of ESKD were excluded from the analysis. Another cohort study by Chen et al. involving 12,778 critically ill adult patients demonstrated that venous congestion, characterized by either peripheral edema or elevated CVP, was directly linked to AKI. Each 1 cm H_2_O increase in CVP was associated with a 2% higher risk of AKI (*p*: 0.02), which is consistent with our findings. We reported that each mmHg increase in CVP ≥ 8 mmHg at 6 h and 48 h was related to AKI with an OR: 1.47 (*p*: 0.015) and OR: 1.44 (*p*: 0.006), respectively, but not at 12 h and 24 h (*p*: 0.248 and *p*: 0.055) [15].

Another significant finding from our study was that the percentage of patients with MAP of ≥65 mmHg was not significantly different between those with and without AKI at 6 h. Still, MAP was notably lower in the AKI group at 12, 24, and 48 h. This suggests that the isolated optimization of arterial pressure in septic shock patients did not prevent organ dysfunction and complications, such as AKI.

In our study, patients in the AKI group exhibited significantly higher in-hospital and 90-day mortality rates compared to those without AKI, which suggests that AKI is associated with a poorer prognosis and underscores the severity of this condition in affecting outcomes of sepsis patients. Elevated CVP has been linked to increased mortality in patients with septic shock in some studies, particularly in the range of 12–24 h [24,27]. These findings suggest that CVP can be a useful predictor of AKI and may be utilized to avoid fluid overload within the first 12–24 h of septic shock [27]. Moreover, our analysis revealed that CVP is generally not a reliable predictor of mortality when controlling clinical variables at different time points. However, an exception was noted for CVP measured at 12 h, which was associated with increased in-hospital mortality before and after adjustments for MAP. This association was insignificant after further adjusting for AKI and other clinical variables. Therefore, the initial observed effect of CVP on mortality may be confounded by the presence of AKI and other clinical factors. When evaluating prognosis in patients with sepsis or septic shock, it is crucial to consider a broader range of clinical characteristics. Recently, this comprehensive evaluation has been enhanced by using machine learning models, which can analyze numerous predictors from large datasets to improve prognostic accuracy in sepsis patients [28,29,30].

Huang et al. [31] analyzed 1986 critically ill patients with AKI and showed an association between high CVP and 90-day mortality, including patients diagnosed with AKI within 48 h. This suggests that the severity of AKI may be a more significant predictor of poor outcomes than elevated CVP. To minimize selection bias, we excluded patients with a known history of ESKD or existing AKI at the time of ICU admission, specifically those diagnosed within 24 h, as well as patients who were discharged or died within 48 h of ICU admission.

This study’s retrospective design poses several limitations. First, we assume that the standard of care was followed in the insertion and maintenance of the central venous catheter. Secondly, we assume that the measurement of CVP was performed as accurately as possible, permitted by the clinical condition of the patient by a trained professional. Third, the study design may cause selection bias and limit the ability to establish a causal relationship between CVP and AKI. Fourth, the type and origin of infection, the species of microorganism in a positive culture, the source control and timing of antibiotic therapy, the use of diuretics before AKI onset, and the known history of malignancy were not considered in this study [32,33,34,35]. These factors can influence patient outcomes and should be considered in future studies.

## 5. Conclusions

In conclusion, we found that CVP levels and the frequency of elevated CVP (≥8 mmHg) were consistently higher in the AKI groups across all measured time points (6, 12, 24, and 48 h following sepsis diagnosis). Elevated CVP was strongly associated with an increased risk of AKI at 12 and 48 h. These findings may provide a modifiable risk factor within time restrictions to reduce the incidence of AKI among patients with septic shock. Identifying sub-phenotypes of ICU patients with conditions that can change the CVP, e.g., the pressure of positive airway pressure, or its association with AKI, e.g., acuity or chronicity of elevated CVP, is an essential next step. Following sub-phenotype adjudications, identifying a CVP threshold that is associated with a higher risk of AKI in each sub-cohort is also necessary.

## Figures and Tables

**Table 1:** Basic characteristics of all patients and comparative analysis between patients with and without AKI.

	Total (3128)	AKI (1098)	No AKI (2030)	*p*-Value
**Sex (Male %)**	1681 (53.74)	679 (61.83)	1002 (49.35)	<0.001
**Age (Median [IQR])**	65.54 [54.63–75.52]	65.24 [54.97–74.76]	65.63 [54.49–75.91]	0.313
**BMI (Median [IQR])**	27.52 [23.45–32.64]	28.73 [24.39–34.42]	26.94 [22.89–31.68]	<0.001
**Hypertension (%)**	2180 (69.69)	791 (72.04)	1389 (68.42)	0.036
**Diabetes Mellitus (%)**	795 (25.41)	300 (27.32)	495 (24.38)	0.072
**Myocardial Infarction (%)**	340 (10.87)	106 (9.65)	234 (11.52)	0.108
**Congestive Heart Failure (%)**	369 (11.79)	144 (13.11)	225 (11.08)	0.093
**Diastolic Heart Failure (%)**	319 (10.19)	132 (12.02)	187 (9.21)	0.013
**Pulmonary Hypertension (%)**	504 (16.11)	180 (16.39)	324 (15.96)	0.753
**Cerebrovascular accidents (%)**	266 (8.50)	66 (6.01)	200 (9.85)	<0.001
**Chronic Kidney Disease (%)**	461 (14.73)	176 (16.02)	285 (14.04)	0.134
**Baseline Cr (Median [IQR])**	0.90 [0.75–1.10]	0.90 [0.74–1.06]	0.93 [0.75–1.10]	<0.001
**Baseline eGFR (Median [IQR])**	72.93 [53.86–99.24]	81.34 [57.57–102.05]	70.57 [52.45–97.17]	<0.001
**Cr Closest to ICU Admission (Median [IQR])**	1.00 [0.70–1.40]	1.40 [1.00–2.02]	0.80 [0.60–1.10]	<0.001
**Max Cr before AKI (Median [IQR])**	1.60 [1.30–2.10]	1.60 [1.30–2.20]	1.30 [1.10–1.80]	<0.001
**APACHE III Score SAS 1 h (Median [IQR])**	51 [39–65]	53 [40–69]	50 [38–64]	<0.001
**APACHE III Score SAS 24 h (Median [IQR])**	80 [65–97]	90 [74–106]	75 [61–91]	<0.001
**Dobutamine 24 h (%)**	146 (4.66)	60 (5.46)	86 (4.23)	0.120
**Dopamine 24 h (%)**	126 (4.03)	46 (4.20)	80 (3.94)	0.736
**Epinephrine 24 h (%)**	180 (5.75)	98 (8.92)	82 (4.04)	<0.001
**Norepinephrine 24 h (%)**	1594 (51.00)	608 (55.37)	986 (48.57)	<0.001
**Phenylephrine 24 h (%)**	486 (15.54)	188 (17.12)	298 (14.70)	0.072
**Vasopressin 24 h (%)**	909 (29.06)	380 (34.61)	529 (26.06)	<0.001
**WBC closest to ICU admission**	10.70 [6.40–16.20]	10.45 [5.77–16.72]	10.80 [6.60–16.10]	0.198
**Max WBC closest to AKI (Median [IQR])**	12.70 [7.50–18.90]	12.60 [7.30–18.80]	13.40 [8.70–19.10]	0.253
**Lactate closest to ICU admission (Median [IQR])**	1.80 [1.10–3.10]	2.00 [1.30–3.60]	1.70 [1.10–2.80]	<0.001
**Net Fluid Balance 24 h (Median [IQR])**	4547.64 [2139.03–7345.28]	4833.82 [2214.10–7838.27]	4382.44 [2063.83–7101.68]	0.002
**Mechanical ventilation (%)**	2324 (74.3)	849 (77.3)	1475 (72.7)	0.005
**Average PEEP (Median [IQR])**	6.86 [5.03–9.15]	7.24 [5.37–9.47]	6.61 [5.00–8.96]	<0.001
**In-ICU Dialysis (%)**	360 (11.5)	315 (28.7)	45 (2.2)	<0.001
**In-Hospital Dialysis (%)**	416 (13.3)	347 (31.6)	69 (3.4)	<0.001
**Length of Hospital Stay**	16.24 [9.66–28.36]	20.60 [12.89–35.15]	14.22 [8.73–24.55]	<0.001
**28-day mortality (%)**	684 (21.90)	345 (31.42)	339 (16.70)	<0.001
**90-day mortality (%)**	977 (31.23)	454 (41.35)	523 (25.80)	<0.001

Data are presented as number (percentage) or median [interquartile range, IQR]. Abbreviations: BMI: body mass index, eGFR: estimated glomerular filtration rate, Cr: creatinine, APACHE: Acute Physiology and Chronic Health Evaluation, WBC: white blood cell, PEEP: positive end-expiratory pressure.

**Table 2:** Comparative analysis of CVP, MAP, and MPP between patients with and without AKI at 4 time points after diagnosis of sepsis.

	Total (3128)	AKI (1098)	No AKI (2030)	*p*-Value
**After 6 h**				
**CVP 6 h (Median [IQR])**	9 [5–13]	10 [6–15]	8 [4–12]	<0.001
**MPP 6 h (Median [IQR])**	62 [54–72]	60 [52–68]	63 [56–74]	<0.001
**CVP 6 h** ≥ **8 (%)**	1189 (38.01)	445 (40.52)	744 (36.65)	<0.001
**MAP 6 h (Median [IQR])**	71 [63–80]	70 [63–79]	71 [64–81]	0.034
**MAP 6 h** ≥ **65 (%)**	2208 (70.58)	756 (68.85)	1452 (71.53)	0.111
**After 12 h**				
**CVP 12 h (Median [IQR])**	8 [4–13]	10 [6–14]	7 [4–12]	<0.001
**MPP12 h (Median [IQR])**	63 [55–73]	60 [52–70]	65 [57–74]	<0.001
**CVP 12 h** ≥ **8 (%)**	1290 (41.24)	511 (46.54)	779 (39.36)	<0.001
**MAP 12 h (Median [IQR])**	72 [65–81]	70 [63–79]	72 [65–82]	<0.001
**MAP 12 h** ≥ **65 (%)**	2346 (75.00)	777 (70.76)	1569 (77.29)	<0.001
**After 24 h**				
**CVP 24 h (Median [IQR])**	9 [5–13]	10 [6–15]	8 [4–12.75]	<0.001
**MPP 24 h (Median [IQR])**	64 [56–74]	61 [53–71]	66 [57–75]	<0.001
**CVP 24 h** ≥ **8 (%)**	1543 (49.33)	616 (56.25)	927 (45.66)	<0.001
**MAP 24 h (Median [IQR])**	73 [65–83]	72 [64–81]	74 [65–84]	<0.001
**MAP 24 h** ≥ **65 (%)**	2443 (78.10)	817 (74.41)	1626 (80.10)	<0.001
**After 48 h**				
**CVP 48 h (Median [IQR])**	9 [5–14]	10 [6–15]	8 [4–13]	<0.001
**MPP 48 h (Median [IQR])**	67 [58–79]	64 [55–74]	68 [60–80]	<0.001
**CVP 48 h** ≥ **8 (%)**	1660 (53.70)	684 (62.30)	976 (48.08)	<0.001
**MAP 48 h (Median [IQR])**	76 [68–87]	74 [67–85]	77 [68–88]	<0.001
**MAP 48 h** ≥ **65 (%)**	2625 (83.92)	888 (80.87)	1737 (85.56)	<0.001

Data are presented as number (percentage) or median [interquartile range, IQR].

**Table 3:** The relationship between CVP and AKI.

CVP and AKI						
	Unadjusted OR (95% CI)	*p* Value	Adjusted OR (95% CI) [Model 1]	*p* Value	Adjusted OR (95% CI) [Model 2]	*p* Value
CVP 6 h ≥ 8	1.56 (1.30–1.89)	<0.001	1.61 (1.33–1.94)	<0.001	1.20 (0.92–1.57)	0.173
CVP 12 h ≥ 8	1.75 (1.47–2.09)	<0.001	1.81 (1.52–2.16)	<0.001	1.60 (1.26–2.05)	<0.001
CVP 24 h ≥ 8	1.87 (1.58–2.21)	<0.001	1.89 (1.60–2.24)	<0.001	1.25 (0.99–1.57)	0.056
CVP 48 h ≥ 8	1.78 (1.52–2.09)	<0.001	1.78 (1.52–2.09)	<0.001	1.60 (1.29–1.99)	<0.001

Model 1: adjusted for MAP ≥ 65. Model 2: adjusted for MAP ≥ 65, age, sex, BMI, APACHE, comorbidities, vasopressors, baseline Cr, eGFR and lactate, average PEEP, and mechanical ventilation.

**Table 4:** The relationship between CVP and mortality.

CVP and In-Hospital (28-Day) Mortality								
	Unadjusted HR (95% CI)	*p* Value	Adjusted HR (95% CI) (Model 1)	*p* Value	Adjusted HR (95% CI) (Model 2)	*p* Value	Adjusted HR (95% CI) (Model 3)	*p* Value
CVP 6 h ≥8	1.18 (0.97–1.44)	0.090	1.21 (0.99–1.48)	0.051	1.09 (0.90–1.33)	0.346	1.05 (0.81–1.35)	0.704
CVP 12 h ≥8	1.19 (1.00–1.43)	0.046	1.22 (1.02–1.46)	0.029	1.09 (0.92–1.31)	0.303	0.96 (0.76–1.21)	0.739
CVP 24 h ≥8	1.08 (0.92–1.29)	0.325	1.09 (0.93–1.30)	0.285	0.97 (0.82–1.16)	0.792	0.82 (0.66–1.02)	0.075
CVP 48 h ≥8	1.13 (0.96–1.33)	0.126	1.12 (0.96–1.32)	0.147	1.03 (0.87–1.21)	0.724	1.11 (0.91–1.35)	0.315
**CVP and 90-Day Mortality**								
CVP 6 h ≥8	1.17 (0.99–1.37)	0.058	1.20 (1.02–1.41)	0.031	1.10 (0.93–1.30)	0.251	1.08 (0.87–1.34)	0.488
CVP 12 h ≥8	1.12 (0.97–1.30)	0.120	1.14 (0.98–1.32)	0.080	1.05 (0.90–1.22)	0.521	0.92 (0.75–1.11)	0.371
CVP 24 h ≥8	0.99 (0.86–1.14)	0.888	0.99 (0.87–1.14)	0.943	0.91 (0.79–1.04)	0.178	0.82 (0.68–0.98)	0.028
CVP 48 h ≥8	1.06 (0.93–1.21)	0.354	1.06 (0.93–1.21)	0.402	0.98 (0.86–1.12)	0.818	1.06 (0.89–1.25)	0.518

Model 1: adjusted for MAP ≥ 65. Model 2: adjusted for AKI. Model 3: adjusted for MAP ≥ 65, AKI, age, sex, BMI, APACHE, comorbidities, vasopressors, baseline Cr, eGFR and lactate, average PEEP, and mechanical ventilation.

## Data Availability

Limited deidentified data would be available as per request.

## Supplementary Materials

The following supporting information can be downloaded at: https://www.mdpi.com/article/10.3390/jcm14093027/s1, Figure S1: AKI stages and 28-day mortality; Figure S2: AKI stages and 90-day mortality.
