# Supplementary figures and images for: The Association Between Central Venous Pressure and Acute Kidney Injury Development in Patients with Septic Shock
Source: J Clin Med. 2025 Apr 27;14(9):3027. doi: 10.3390/jcm14093027 (PMC12072389; doi:10.3390/jcm14093027)

Supplementary Materials:

Supplementary Figure S1:

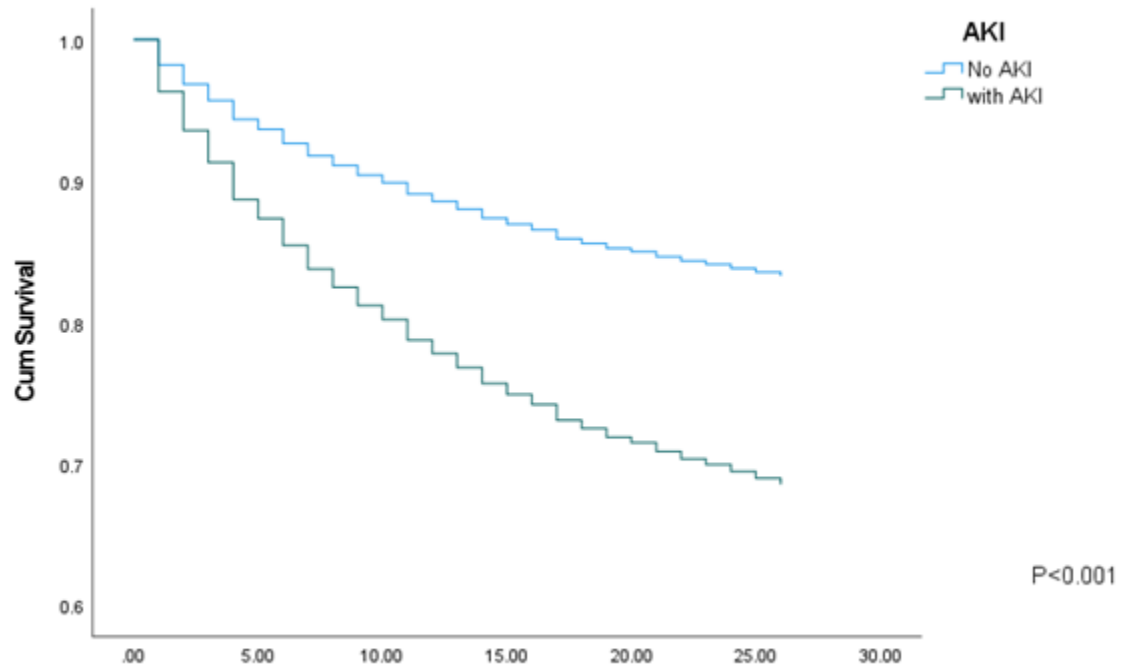

Supplementary Figure S2:

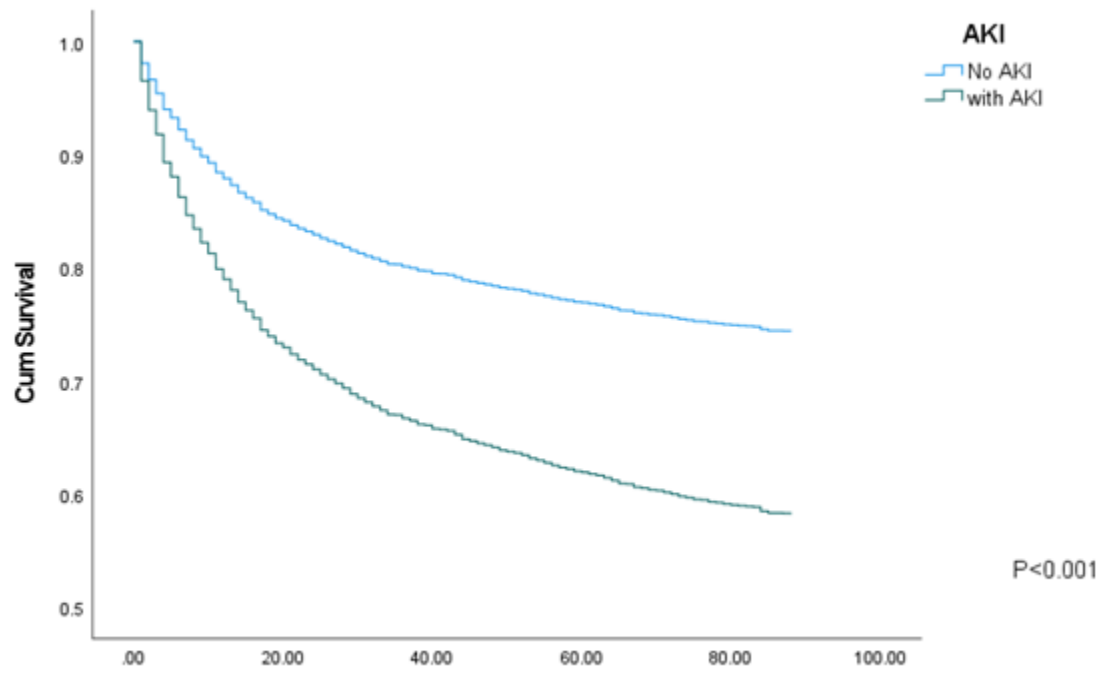

Supplement: Supplementary file 1 [file jcm-14-03027-s001.zip › jcm-3557871-supplementary.pdf]
